# Micronutrients are associated with endoscopic postoperative recurrence in Crohn’s disease: a multicenter prospective cohort study in North America

**DOI:** 10.1093/ecco-jcc/jjaf148

**Published:** 2025-08-20

**Authors:** Michiel T J Bak, Karen Boland, Shadi Nayeri, Krzysztof Borowksi, Pablo A Olivera, Cristian Hernandez-Rocha, Williams Turpin, Joanne M Stempak, Steven R Brant, Judy H Cho, Richard H Duerr, Talin Haritunians, Mark G Lazarev, Emebet Mengesha, Dermot P B McGovern, John D Rioux, L Philip Schumm, Sun-Ho Lee, Mark S Silverberg, the NIDDK Inflammatory Bowel Disease Genetics Consortium, Michiel T J Bak, Karen Boland, Shadi Nayeri, Krzysztof Borowksi, Pablo A Olivera, Cristian Hernandez-Rocha, Williams Turpin, Joanne M Stempak, Steven R Brant, Judy H Cho, Richard H Duerr, Talin Haritunians, Mark G Lazarev, Emebet Mengesha, Dermot P B McGovern, John D Rioux, L Philip Schumm, Sun-Ho Lee, Mark S Silverberg

**Affiliations:** Zane Cohen Centre for Digestive Diseases, Mount Sinai Hospital, Toronto, Canada; Lunenfeld-Tanenbaum Research Institute, Mount Sinai Hospital, Toronto, Canada; Department of Gastroenterology and Hepatology, Erasmus University Medical Center Rotterdam, Rotterdam, the Netherlands; Zane Cohen Centre for Digestive Diseases, Mount Sinai Hospital, Toronto, Canada; Lunenfeld-Tanenbaum Research Institute, Mount Sinai Hospital, Toronto, Canada; Department of Gastroenterology, Beaumont Hospital, Dublin, Ireland; Zane Cohen Centre for Digestive Diseases, Mount Sinai Hospital, Toronto, Canada; Lunenfeld-Tanenbaum Research Institute, Mount Sinai Hospital, Toronto, Canada; Zane Cohen Centre for Digestive Diseases, Mount Sinai Hospital, Toronto, Canada; Lunenfeld-Tanenbaum Research Institute, Mount Sinai Hospital, Toronto, Canada; Zane Cohen Centre for Digestive Diseases, Mount Sinai Hospital, Toronto, Canada; Lunenfeld-Tanenbaum Research Institute, Mount Sinai Hospital, Toronto, Canada; Zane Cohen Centre for Digestive Diseases, Mount Sinai Hospital, Toronto, Canada; Lunenfeld-Tanenbaum Research Institute, Mount Sinai Hospital, Toronto, Canada; Department of Gastroenterology, School of Medicine, Pontificia Universidad Católica de Chile, Santiago, Chile; Zane Cohen Centre for Digestive Diseases, Mount Sinai Hospital, Toronto, Canada; Lunenfeld-Tanenbaum Research Institute, Mount Sinai Hospital, Toronto, Canada; Zane Cohen Centre for Digestive Diseases, Mount Sinai Hospital, Toronto, Canada; Lunenfeld-Tanenbaum Research Institute, Mount Sinai Hospital, Toronto, Canada; Rutgers Crohns and Colitis Center of New Jersey, Department of Medicine, Robert Wood Johnson Medical School, New Brunswick and Piscataway, NJ, United States; Department of Genetics, School of Arts and Sciences, Rutgers, The State University of New Jersey, New Brunswick and Piscataway, NJ, United States; Division of Gastroenterology and Hepatology, Harvey M. and Lyn P. Meyerhoff Inflammatory Bowel Disease Center, Johns Hopkins University School of Medicine, Baltimore, MD, United States; The Charles Bronfman Institute for Personalized Medicine, Icahn School of Medicine at Mount Sinai Hospital, New York, NY, United States; Department of Gastroenterology, University of Pittsburgh, Pittsburgh, PA, United States; The F. Widjaja Inflammatory Bowel Disease Institute, Cedars-Sinai Medical Center, Los Angeles, CA, United States; Division of Gastroenterology and Hepatology, Harvey M. and Lyn P. Meyerhoff Inflammatory Bowel Disease Center, Johns Hopkins University School of Medicine, Baltimore, MD, United States; The F. Widjaja Inflammatory Bowel Disease Institute, Cedars-Sinai Medical Center, Los Angeles, CA, United States; The F. Widjaja Inflammatory Bowel Disease Institute, Cedars-Sinai Medical Center, Los Angeles, CA, United States; Faculty of Medicine, Université de Montréal, Montreal, Canada; Public Health Sciences, University of Chicago, Chicago, IL, United States; Zane Cohen Centre for Digestive Diseases, Mount Sinai Hospital, Toronto, Canada; Lunenfeld-Tanenbaum Research Institute, Mount Sinai Hospital, Toronto, Canada; Zane Cohen Centre for Digestive Diseases, Mount Sinai Hospital, Toronto, Canada; Lunenfeld-Tanenbaum Research Institute, Mount Sinai Hospital, Toronto, Canada

**Keywords:** diet, nutrients, Crohn’s disease, postoperative recurrence

## Abstract

**Background and Aims:**

Diet may influence the disease course in inflammatory bowel disease, but its role in postoperative outcomes for Crohn’s disease (CD) remains unclear. This study aimed to assess the association of macro- and micronutrient intake with endoscopic postoperative recurrence (ePOR) in a prospective multicenter cohort.

**Methods:**

Patients with CD following ileocolic resection were prospectively recruited from six North American centers. Primary study outcome was ePOR (modified Rutgeerts’ score ≥i2a) during follow-up. Nutritional intake was assessed through 2-day food diaries verified by dietitian-delivered interview. Associations between nutrient intake and ePOR were evaluated. Random forest models with 10-fold cross-validation assessed the predictive value of nutrients and clinical factors for ePOR.

**Results:**

A total of 520 food diaries from 103 patients were analyzed; 37 patients (36%) experienced ePOR. Univariate analysis identified 8 nutrients associated with ePOR including lower intake of isoflavones (genistein, daidzein, glycitein; *P *< .01), inositol (*P* < .01), pinitol (*P* = .02), provitamin-A carotenoid (*P* < .01), xylitol (*P *= .03) and parinaric Acid (*P *= .03). Sensitivity analysis confirmed the association of lower intake of all isoflavones (*P *< .01) and pinitol (*P *= .04) with ePOR at first postoperative colonoscopy. Random forest models showed poor discrimination for clinical factors alone (area under the curve [AUC] 0.59) but an acceptable discrimination for nutrients alone (AUC 0.67), which improved when combining nutrients and clinical factors (AUC 0.71).

**Conclusion:**

Lower intake of specific micronutrients is associated with ePOR in CD patients. A machine-learning model combining nutrient intake and clinical factors enhanced the prediction of ePOR. These findings highlight the importance of postoperative nutritional assessment and suggest dietary interventions may help prevent postoperative recurrence in CD.

## 1. Introduction

The pathogenesis of Crohn’s disease (CD) is multifactorial, involving a complex interplay between abnormal innate immune responses, genetic predisposition, and environmental factors, with diet likely playing a central role by supporting physiological processes and influencing the intestinal microbiome.^1^ An important role of diet in CD development is supported by large population-based cohort studies, reporting associations between increased intake of ultra-processed foods, Western-dietary patterns (ie, low fruit and vegetable consumption), and high sucrose intake with a higher risk of CD development.^2–4^ Conversely, adherence to the Mediterranean diet, high fruit and vegetable consumption, or long-term fiber intake has been associated with a reduced risk of CD development.^5–8^

Several diets have been evaluated in patients with mild-to-moderate CD, primarily focusing on their ability to induce clinical remission.^9^ Despite the interest of dietary interventions to treat active CD, the potential impact of diet on the postoperative course of CD remains unknown. As a substantial proportion of patients with CD will undergo an intestinal resection during their disease course, and postoperative disease recurrence is a common clinical dilemma, understanding the role of diet in this setting is critical.^10^^,^^11^ Moreover, patients with CD following intestinal resection may be considered in the “deepest” remission after removal of the affected segment, offering the most homogeneous CD population possible to study the impact of diet on the disease course. This study utilizes data from a previously published prospective multicenter cohort study where clinical and microbiota outcomes were reported.^11^^,^^12^ Building on these findings, in this study we specifically examine the relationship between macro- and micronutrient intake and endoscopic postoperative recurrence (ePOR) in this well-characterized patient population.

## 2. Methods

### Study design

Patients with CD undergoing ileocolic (re-)resection (ICR) with primary anastomosis were recruited during the perioperative period across the 6 North American centers participating in the Genetics Research Centers of the NIDDK Inflammatory Bowel Disease Genetics Consortium (IBDGC).^11^ Patients, with a confirmed diagnosis of CD with ileal involvement based on surgical pathology, were included. Exclusion criteria comprised ileal resection with ileal–ileal anastomosis leaving an intact ileocecal valve, a sub-total or near sub-total colonic resection, resection with temporary or permanent diverting ileostomy, or more than two prior surgeries. The use of postoperative prophylactic medication and the timing of the postoperative ileocolonoscopies were at the discretion of the treating physician. As the use of antibiotics was not considered as postoperative prophylactic medication due to the lack of supporting data, patients were excluded.^13^

### Nutrient analysis

**Figure 1.**
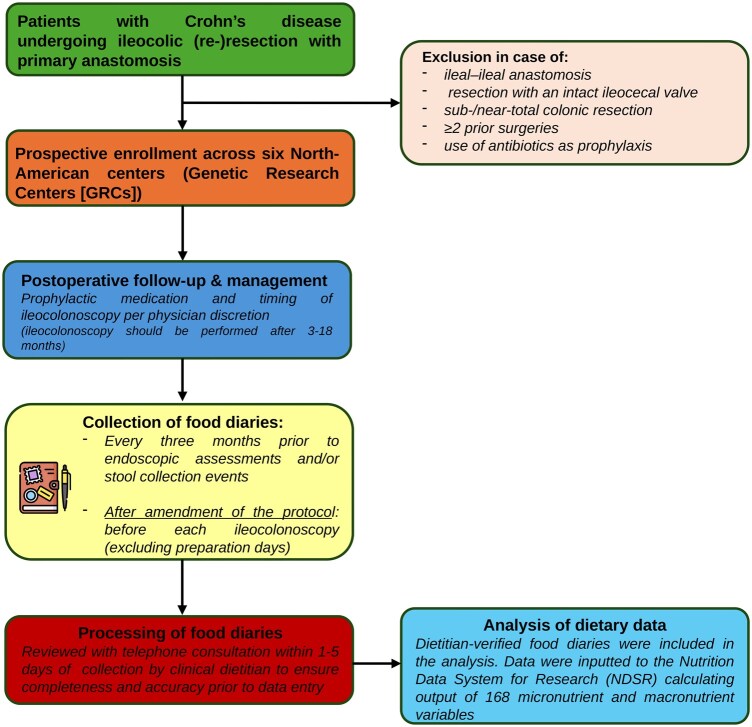
Visual representation of the study design.

A flowchart of the study is presented in Figure 1. Patients were invited to complete 2-day food diaries at recruitment and throughout follow-up, including intake records of vitamin supplements, added sweeteners, and beverages. Initially, patients were invited to complete food diaries every 3 months prior to endoscopic assessments and/or stool collection events. The protocol was amended during the course of the study to request food diaries at specific time points: post-operatively and before each post-operative colonoscopy (excluding preparation days). Patients were invited to complete 2-day food diaries at multiple time points to reduce the likelihood of dietary choices influenced by symptoms. A clinical dietitian conducted a telephone interview to review each food diary within 1-5 days of collection, ensuring completeness and accuracy prior to data entry. Results were inputted to the Nutrition Data System for Research (NDSR), a dietary analysis program which collects and analyses food captured via diaries, calculating output of 168 micronutrient and macronutrient variables.^14^ Only patients with dietitian-verified food diaries were included in the analysis.

### Outcomes

The outcome of the study was ePOR, defined as a modified Rutgeerts’ score ≥i2a, during the study period. The main objective was to investigate the association between nutrient intake and the occurrence of ePOR in patients following ICR for CD.

### Statistical analysis

Statistical analysis was performed using Stata (Statcorp) and R statistical packages. Univariate analysis was performed to identify individual nutrients associated with ePOR during the study period. For this analysis, all available individual food diaries were used to calculate the median value of the individual nutrients per patient. Subsequently, data were quantitatively corrected for energy consumption using body mass index (BMI) and the log-corrected median value of the individual nutrients were compared, using *t*-tests, between patients who developed recurrence and patients who did not develop recurrence. To limit potential confounding factors for both dietary intake and development of ePOR and to account for the timing of the food diary in relation to endoscopic outcomes, a sensitivity analysis was performed for the occurrence of ePOR at first postoperative ileocolonoscopy only using the available food diaries prior to first postoperative ileocolonoscopy. No corrections for multiple testing were made.

Random forest (RF) models were tested with nutrient and clinical variables, both separately and combined, to predict ePOR. These analyses were performed using Python (3.8.20) and scikit-learn (1.3.2). Three RF models were tested: the log-corrected significant nutrient medians from the main analysis, relevant clinical factors for ePOR *a priori* identified based on previous literature and consensus amongst the study group (male sex, active smoking, prior small bowel resection, disease duration and age at surgery, penetrating disease at surgery, perianal disease at surgery, and postoperative prophylactic medication), and finally, the significant nutrients and relevant clinical factors combined.^15^^,^^16^ Each RF model was cross-validated with stratified k-folds (with k = 10), and the receiver operating characteristic (ROC) and area under the curve (AUC) were calculated per fold, along with standard deviations. These values were then averaged to obtain a mean ROC AUC.

## 3. Results

Between May 2014 and July 2019, 103 patients (51% female) completed a total of 520 food diaries (median food diaries 2, interquartile range [IQR] 1-3) (Table 1). Median disease duration and mean age at surgery was 9.0 years (IQR 3.0-15.0) and 34.2 years (standard deviation [SD] 12.7). Active smoking at surgery was reported in 14% (*n* = 14) and 23% (*n* = 24) of patients underwent a prior resection. Median time to first postoperative endoscopy was 7.7 months (IQR 5.6-9.2). ePOR was diagnosed in 36% (*n* = 37) during the study period. The mean follow-up duration of the study cohort was 2.3 years (SD 1.6). No significant differences in baseline characteristics were observed between patients who were diagnosed with ePOR as compared to patients with no diagnosis of ePOR.

**Table 1.** Description of the study population.

Variable	Total study population (*n* = 103)	Patients in endoscopic remission (i0-i1) (*n* = 66)	Patients with endoscopic recurrence (≥i2a) (*n* = 37)	*P*-value
Sex (female), *n(%)*	52 (51)	30 (46)	22 (60)	0.25
Age at surgery (years), *mean (SD)*	34.2 (12.7)	32.8 (11.0)	36.6 (15.2)	0.14
Active smoker, *n(%)*	14 (14)	7 (11)	7 (19)	0.50
* Missing*	3 (3)	2 (3)	1 (3)	
Prior resection, *n(%)*	24 (23)	14 (21)	10 (27)	0.67
Montreal disease location at surgery, *n(%)*				0.44
*L1*	36 (35)	26 (39)	10 (27)	
*L3*	64 (62)	38 (58)	26 (70)	
* Missing*	3 (3)	2 (3)	1 (3)	
Montreal disease behavior at surgery, *n(%)*				0.38
*B1*	2 (2)	2 (3)	0	
*B2*	44 (43)	30 (46)	14 (38)	
*B3*	57 (55)	34 (51)	23 (62)	
Perianal involvement (p+), *n(%)*	33 (32)	19 (29)	14 (38)	0.27
* Missing*	5 (5)	2 (3)	3 (8)	
Treatment at first endoscopy, *n(%)*	61 (59)	42 (64)	19 (51)	0.32
* Immunomodulator (thiopurines and/or methotrexate)*	6 (6)	2 (3)	4 (11)	0.24
* Anti-tumor necrosis factor agents*	22 (21)	16 (24)	6 (16)	0.48
* Ustekinumab*	11 (11)	6 (9)	5 (14)	0.72
* Vedolizumab*	2 (2)	1 (2)	1 (3)	1.00
*Combination therapy (immunomodulator and biological)*	20 (19)	17 (26)	3 (8)	0.06
Time to endoscopy (months), *median* (*IQR*)	7.7 (5.6–9.2)	7.8 (5.9–9.4)	6.6 (5.0–8.9)	0.17
Time to endoscopic recurrence (months), *median (IQR)*	-	-	8.5 (6.5–18.5)	-

Abbreviations: IQR, interquartile range; SD, standard deviation.

### Univariate analysis for the identification of nutrients with endoscopic postoperative recurrence

**Figure 2.**
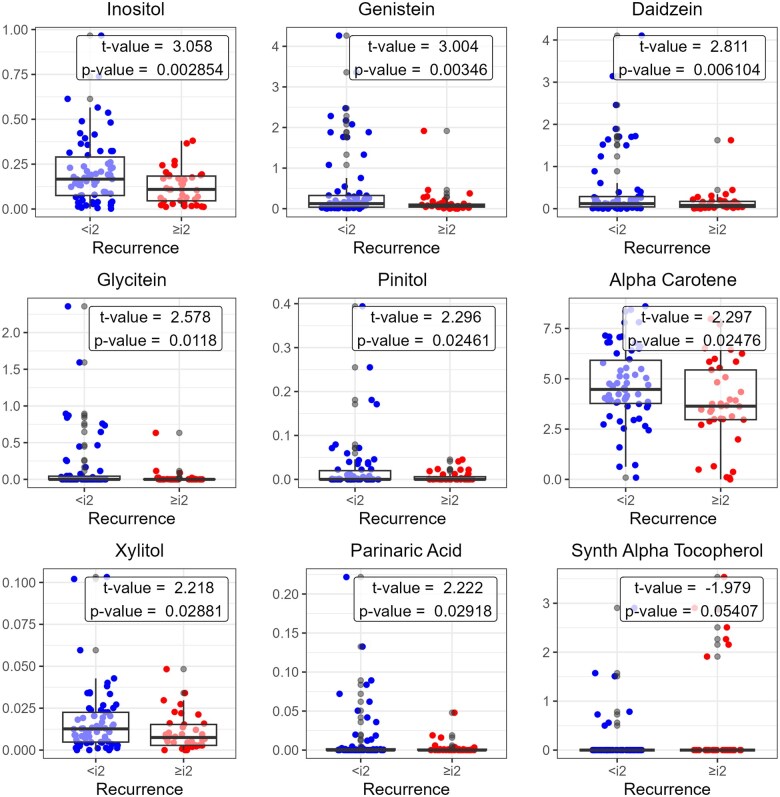
Univariate analysis with the top 9 nutrients associated with endoscopic recurrence (modified Rutgeerts’ score ≥i2a) during the study period. * y-axis represents the log value of the nutrients

**Figure 3.**
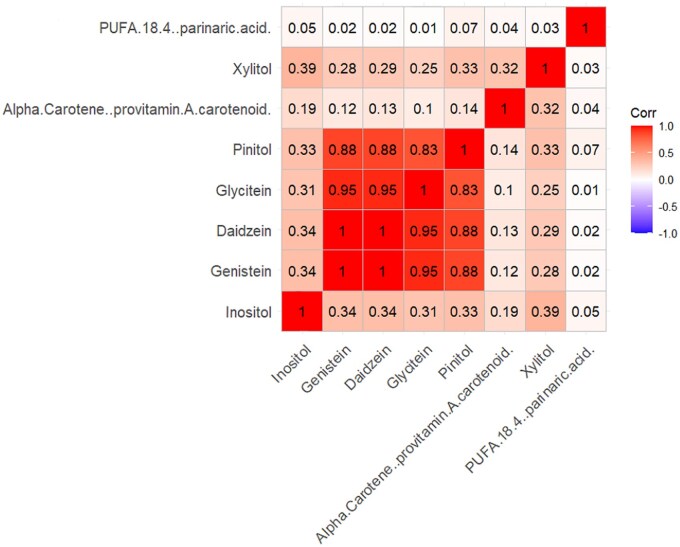
Correlation plot of the significant nutrients.

Nutrient analysis of all assessed macro- and micronutrients (both median and mean log values) are presented in Table S1 and S2. No statistically significant difference in total calorie intake (*P* = .74) or weight gain (*P* = .58) according to presence or absence of ePOR was observed. Univariate analysis identified 8 nutrients found to be associated with ePOR during the study period (Figure 2). Specifically, intake of genistein (*P *< .01), daidzein (*P *< .01), glycitein (*P *< .01), inositol (*P *< .01), pinitol (*P *= .02), provitamin-A carotenoid (*P *< .01), xylitol (*P *= .03), and parinaric acid (*P *= .03) were all significantly lower in patients who were diagnosed with ePOR. As expected, correlation between the isoflavones (glycitein, daidzein, and genistein) was very strong (*r* = 1) (Figure 3). Except for a strong to very strong correlation between pinitol and isoflavones (glycitein [*r* = 0.83], daidzein [*r* = 0.88], and genistein [*r* = 0.88]), a weak correlation (*r* < 0.4) was observed between the other significant nutrients.

**Figure 4.**
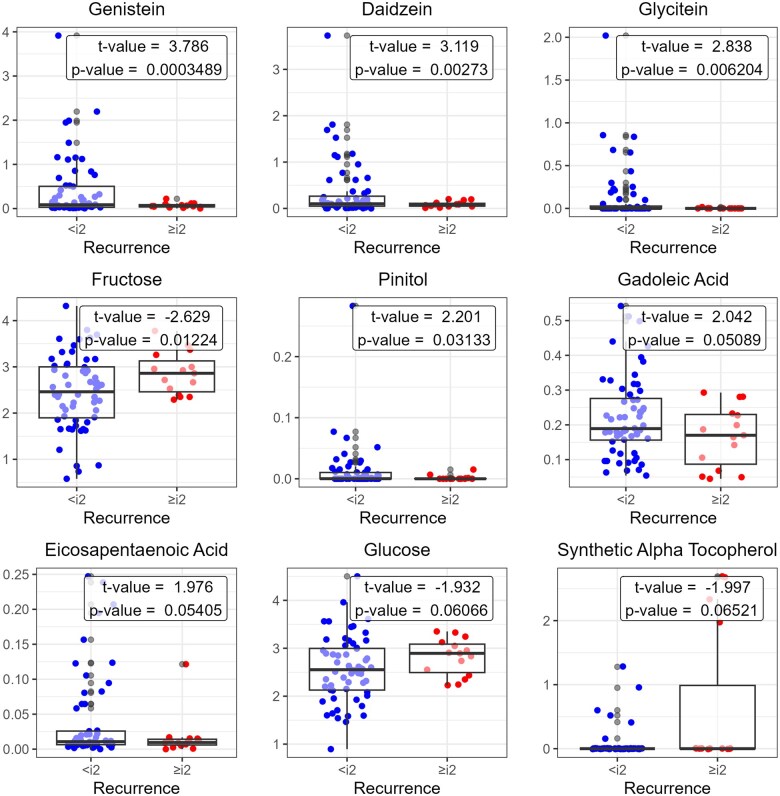
Univariate analysis with the top 9 nutrients associated with endoscopic recurrence (modified Rutgeerts’ score ≥i2a) at first postoperative ileocolonoscopy. * y-axis represents the log value of the nutrients

Sensitivity analysis was performed in patients with available food diaries prior to first postoperative ileocolonoscopy (*n* = 75, 73%), which confirmed the associations of lower intake of isoflavones (genistein, daidzein, and glycitein; *P *< .01) and pinitol (*P *= .03) with ePOR at first postoperative ileocolonoscopy (Figure 4). In addition, an association was found for higher intake of fructose (*P *= .01).

### Random forest models

**Figure 5.**
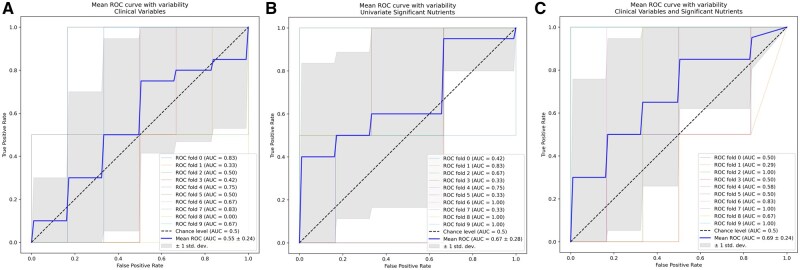
Random forest models for clinical factors (*left*), identified significant nutrients in univariate analysis (*middle*), and combined model with clinical risk factors and identified significant nutrients (*right*). Abbreviations: ROC, receiver operating curve; clinical factors comprised male sex, active smoking, prior bowel resection, disease duration at surgery, age at surgery, penetrating disease, and perianal disease at surgery.

The random forest models are displayed in Figure 5A-C. The mean ROC curve of the clinical factors alone was 0.59 (SD 0.24), whereas the mean ROC curve for the identified associated nutrients was 0.67 (SD 0.28). The performance of the combined model (clinical factors and identified associated nutrients) improved to a mean ROC of 0.71 (SD 0.20). Interestingly, 7 out of the top 10 contributors to the combined model were nutrients (age at surgery [0.129], provitamin-A carotenoid [0.121], xylitol [0.108], inositol [0.107], disease duration at surgery [0.102], genistein [0.093], daidzein [0.090], parinaric acid [0.073], active smoking at surgery [0.040], pinitol [0.032]). Details on the variable importance for each model are reported in Table S3.

## 4. Discussion

To the best of our knowledge, this study is the first to assess the potential impact of both macro- and micronutrients on the postoperative disease course in CD. Our findings revealed a significantly lower intake of isoflavones (genistein, daidzein, glycitein), inositol (vitamin B8), pinitol (a cyclitol), provitamin-A carotenoid, xylitol (natural sweetener), and parinaric acid (polyunsaturated fatty acid) in patients with CD who developed ePOR throughout the study period. A strong correlation was observed among the isoflavones, as well as between pinitol and isoflavones, whereas correlations between other nutrients were generally weak. Sensitivity analyses confirmed that lower intake of isoflavones and pinitol was associated with an increased risk of ePOR at the first postoperative colonoscopy. In addition, higher intake of fructose (a monosaccharide) was associated with the occurrence of ePOR. Furthermore, a machine-learning based model combining nutrient intake with clinical variables increased the performance to predict ePOR as compared to clinical factors or nutrients alone. Our results suggest that diet may influence the postoperative disease course in patients with CD.

The literature on the impact of pre- or postoperative diet on the course of postoperative CD is limited.^17–23^ A recently published randomized controlled trial from China reported significant lower ePOR rates at both 3 (*P *< .01) and 12 months (*P *= .04) in patients treated with azathioprine and 3-months exclusive enteral nutrition (EEN) as compared to patients treated with azathioprine alone. However, quality of life was significantly higher in the azathioprine-only group at 3 months but comparable between 5 and 12 months postoperatively.^17^ Another Chinese randomized trial found that preoperative EEN combined with azathioprine improved nutritional blood markers (albumin and CRP) and clinical symptoms, but no difference in ePOR at 12 months between groups focused on malnutrition improvement versus inflammation reduction.^22^ Other observational studies on preoperative EEN (3 studies) and postoperative elemental diets (2 studies) yielded conflicting results.^18–21^^,^^23^ Despite the great efforts of these study groups, the outcomes of these studies are difficult to interpret as these studies are predominantly non-randomized, single-center studies with small sample sizes, varying regimens, and treatment durations.^17–23^ Therefore, larger, preferably randomized, studies are warranted to further explore the role of diet in the postoperative course in CD.

It is hypothesized that postoperative recurrence in CD is dependent on the continuity of the fecal stream which contributes to the role of the microbiome in the development of disease recurrence after surgery.^24^ A recent systematic review reported associations of several individual bacterial taxa, found in the resection specimen, neoterminal ileum, or feces, with (severe) ePOR.^15^ In addition, an acceptable discrimination of 2 separate models was reported, including microbial factors in the neoterminal ileum, to predict ePOR within 1 year postoperatively (AUC 0.74-0.81).^25^^,^^26^ The study by Hernández-Rocha *et al.* investigated whether the ileal biopsy-associated microbiota was associated with disease progression in patients with endoscopic remission (Rutgeerts’ score i0-i1) at the first postoperative ileocolonoscopy.^12^ A lower diversity and microbial deviations were found in patients who developed ePOR (Rutgeerts’ score ≥i2) as compared to those who were in sustained endoscopic remission at the second postoperative ileocolo-noscopy. In addition, several genera (*Anaerostipes* and *Gammoproteobactericia*) increased the risk of disease recurrence. In line with our findings, their study identified a poor to acceptable discrimination to predict ePOR based on clinical factors alone (AUC 0.56 to 0.68) whereas a combined model (microbial factors and clinical factors) improved the performance of the model (AUC 0.80 to 0.83).^12^ Outcomes of all these studies suggest an important contribution of the microbiome to postoperative disease recurrence development in CD, and identifies a previously unrecognized therapeutic target for preventing recurrence after surgery. Further research is warranted to assess the correlation between dietary patterns or nutrient intake with the microbial composition. Due to variable intervals of food diaries and biopsy collection at the postoperative ileocolonoscopies, as described in our earlier published work, we were not able to study the relationship between nutrient intake and the microbiome.^12^ Evaluation of novel treatment strategies aiming to improve the microbial composition, such as diet, probiotics, and fecal microbial transplantation, is warranted to be examined first in high-quality studies before offering this non-pharmacological treatment option in the postoperative disease course as a treatment strategy or as an add-on to therapeutic regimens.

Our results showed that a lower intake of isoflavones (genistein, daidzein, and glycitein) was consistently associated with ePOR. Isoflavones, a subgroup of flavonoids, are naturally occurring in certain fruits, nuts, soy products, and legumes, and act as exogenous antioxidants with anti-inflammatory effects.^27^^,^^28^ In experimental studies, flavonoids have demonstrated benefits for diseases like inflammatory bowel disease (IBD), rheumatoid arthritis, cardiovascular disease, and cancer.^28^ Isoflavones interact with estrogen receptors, influencing gene expression and reducing intestinal permeability and pro-inflammatory factors.^29^ Experimental (colitis) models show that isoflavones reduce inflammatory factors (IL-6, IL-1 β, and TNF-α), and nitric oxide synthase and prostaglandin E2 production which have a pro-inflammatory effect.^30^^,^^31^ Furthermore, isoflavone consumption increases anti-inflammatory monounsaturated fatty acids (MUFAs) and beneficial polyunsaturated fatty acids (PUFAs), and reduces activity of pro-inflammatory pathways and associated metabolites.^32^ Conflicting effects of isoflavone intake in ulcerative colitis (UC) have been reported, including both a reduced rate of abdominal complaints and an increased occurrence of fecal pus or even disease onset.^33–35^ In addition to the association with isoflavones, pinitol was also consistently linked to ePOR in our study. Pinitol, a cyclitol found in soybeans, legumes, and seeds, was strongly to very strongly associated with isoflavones as expected as both nutrients (isoflavones and pinitol) are found in soy products and legumes. Pinitol has been recognized as an anti-diabetic agent with an antioxidant effect.^36^ In addition, an association was found for inositol, a sugar alcohol found in animal protein, legumes, seeds, and nuts, with anti-diabetic, anti-oxidant, and anti-inflammatory effects.^37^ As lower intake of these nutrients has also been associated with ePOR in our study, and these nutrients share dietary sources with the isoflavones, the outcomes reinforce the potential association identified between increased intake of legumes and soy products with endoscopic remission in this cohort. The findings support dietary strategies that include legumes, seeds, and other antioxidant-rich foods, such as the Mediterranean diet, as recently recommended by the American guidelines, but also underlines the importance of postoperative dietary counseling by a dietitian as a substantial proportion of patients may unnecessarily restrict these food products/groups.^38^ Further prospective studies are required to identify whether these associations may have therapeutic applications or potential to support dietary recommendations in patients with established CD.

Besides the identification of nutrients predominantly present in soy and legumes, other associations were found with ePOR including a lower intake of provitamin-A carotenoid, xylitol, parinaric acid, and a higher intake of fructose. Disease-related outcomes of carotenoid, the provitamin of Vitamin A with an anti-oxidant effect, in IBD were only studied in 1 multicenter study.^39^ In line with our findings, significantly lower carotenoid levels were measured in patients with active IBD as compared to patients with inactive IBD.^39^ Unfortunately, no definition of active IBD nor inactive IBD was described in this study. For the effects of artificial sweeteners, such as fructose, only preclinical studies have been performed in IBD reporting both beneficial and deleterious effects of artificial sweeteners on gut health, whereas negative effects of high fructose diets were described, including change in gut barrier function and microbial composition/distribution.^40–42^ It is important to note that a detailed analysis of fructose sources was not feasible with the current data. Since fructose can be derived from both health-promoting foods (such as fruits) and less healthy sources (such as processed foods containing high-fructose corn syrup), this distinction has important implications for practical dietary recommendations. Therefore, further research is warranted. The Mediterranean diet, enriched with high ratios of MUFA and omega-3 PUFA, has shown to be associated with decreased risk of subclinical intestinal inflammation or CD development.^5^^,^^43^ Specifically, an increased dietary intake of PUFA (fatty fish, nuts, seeds, and soy products) or PUFA supplementation, especially omega-3 PUFA including parinaric acid, has been associated with a significantly decreased risk of CD development, postoperative complications, and pro-inflammatory cytokines in CD.^44–47^ However, an excess of PUFA in a Western diet may also lead to deterioration of the disease course as it triggers enteric inflammation.^48^ For MUFA intake, inconsistent findings have been described with regards to disease development and outcomes.^49^ No studies have been performed for gadoleic acid specifically and disease outcomes in the IBD population. The impact of these nutrients needs to be further elucidated in high-quality studies as the literature on clinical disease outcomes is either lacking or inconsistent in the IBD population.

This study is the first to assess the impact of both macro- and micronutrients on ePOR in CD. The strengths of our study are the prospective study design, in the most homogeneous cohort possible in CD, with a longitudinal analysis of nutrients using multiple 2-day food diaries followed by clinical dietitian validation and correction for energy intake. This should increase the potential accuracy of the reported dietary associations with endoscopic disease recurrence. We acknowledge that the dietary pattern may vary over time as measured with the 2-day food diaries. To limit potential variability for dietary intake and confounding factors for development of ePOR and to account for timing of the food diary in relation to endoscopic outcomes, we performed a sensitivity analysis using only food diaries completed before the first postoperative ileocolonoscopy which confirmed the associations for several nutrients. Furthermore, we used an objective parameter (ie ePOR) as an endpoint in our study. In addition, we used a machine-learning based model to explore the predicted probability of the associated nutrients alone or combined with known clinical risk factors for ePOR. Nevertheless, intervals between food diaries were variable due to a lack of stringent patient adherence with this aspect of the protocol and because of the subsequent protocol change during recruitment as outlined in our study methods. Furthermore, although NDSR was used to calculate macro- and micronutrient intake, it does not provide sufficient detail to accurately categorize foods into specific food groups or processing categories, making it challenging to assess dietary intake by food group or processing level directly from the software. As clinical symptom data were routinely not collected alongside dietary assessments, we are unable to determine whether patients were in clinical remission at the time of endoscopy. In order to reduce the likelihood of dietary choices that are influenced by symptoms, patients were invited to complete 2-day food diaries at multiple time points. These variable intervals also did not allow us to study the relationship between nutrient intake and the microbiome or omics analysis such as metagenomics. Therefore, we encourage future research to include these approaches to better understand diet–microbiota interactions. And last, these outcomes may only apply to the North-American population or countries with comparable diets. Due to the lack of a prospective cohort study with available diet data, in the setting of postoperative CD, we are not able to validate our outcomes.

## 5. Conclusion

To our knowledge, this study if the first and only study to prospectively assess dietary components and the association with postoperative recurrence in CD. Our outcomes showed that a lower intake of several micronutrients was associated with endoscopic postoperative recurrence in patients with CD. In addition, we found that combining nutrients with clinical variables as part of a machine-learning based model improved the performance to predict endoscopic postoperative recurrence. These associations suggest the importance of assessing nutritional factors postoperatively in patients with CD and provide a potential basis to design prospective dietary intervention studies for the prevention of postoperative disease recurrence in CD.

## Supplementary Material

jjaf148_Supplementary_Data

## Data Availability

The data underlying this article will be shared on reasonable request to the corresponding author.
