# Supplementary material for: Micronutrients are associated with endoscopic postoperative recurrence in Crohn’s disease: a multicenter prospective cohort study in North America
Source: J Crohns Colitis. 2025 Aug 20;20(6):jjaf148. doi: 10.1093/ecco-jcc/jjaf148 (PMC13365150; doi:10.1093/ecco-jcc/jjaf148)
Supplement: jjaf148_Supplementary_Data [file jjaf148_supplementary_data.zip › Supplemental Material.docx]

**Supplementary Materials**

| **Micro- or macronutrients** | **Median value in no ePOR group** | **Median value in ePOR group** |
| --- | --- | --- |
| kcal | 2243,53 | 2186,109 |
| TotalFat | 82,37225 | 75,56296 |
| TotalCarbohydrate | 247,5056 | 231,6508 |
| TotalProtein | 95,05236 | 77,62859 |
| AnimalProtein | 58,7235 | 56,95449 |
| VegProtein | 27,05576 | 28,08932 |
| Alcohol | 0,017181 | 0,022022 |
| Cholesterol | 258,0898 | 305,6339 |
| SFA | 25,80027 | 26,33093 |
| MUFA | 30,55159 | 27,91975 |
| PUFA | 17,8139 | 17,48368 |
| Fructose | 16,99578 | 18,6317 |
| Galactose | 0,142411 | 0,134777 |
| Glucose | 15,14749 | 16,90637 |
| Lactose | 5,566171 | 4,949565 |
| Maltose | 2,123199 | 1,938126 |
| Sucrose | 42,72823 | 44,12968 |
| Starch | 103,0928 | 94,42892 |
| TotalFiber | 19,44727 | 17,00262 |
| SolFibre | 6,423332 | 5,971709 |
| InsolFibre | 12,07458 | 10,01053 |
| Pectins | 2,637292 | 1,593627 |
| VitAActivity | 4764,326 | 4209,207 |
| BetaCaroteneEquivalents | 1790,75 | 1260,331 |
| Retinol | 490,129 | 428,5207 |
| Calciferol | 4,853589 | 4,27032 |
| AlphaTocopherol | 12,19286 | 12,7708 |
| VitaminE | 10,171 | 10,23448 |
| BetaTocopherol | 0,484184 | 0,405963 |
| GammaTocopherol | 11,66228 | 13,82156 |
| DeltaTocopherol | 2,754879 | 2,585165 |
| VitaminK | 84,01408 | 80,09613 |
| VitaminC | 55,42923 | 62,19732 |
| VitaminB1 | 1,706953 | 1,721 |
| VitamonB2 | 2,249467 | 2,017407 |
| VitaminB3 | 28,1654 | 25,26305 |
| PantothenicAcid | 5,978516 | 5,1019 |
| VitaminB6 | 2,32756 | 1,874674 |
| Folate | 406,6861 | 394,4939 |
| VitaminB12 | 4,231311 | 3,890328 |

**Supplementary Table 1.** Nutrient analysis of all assessed macro- and micronutrients (*median values*)

**Abbreviations**. ePOR = endoscopic postoperative recurrence

| **Micro- or macronutrients** | **Median value in no ePOR group** | **Median value in ePOR group** |
| --- | --- | --- |
| Calcium | 900,4777 | 848,2981 |
| Phosphorus | 1357,736 | 1140,472 |
| Magnesium | 323,9113 | 262,5929 |
| Iron | 15,42187 | 15,39518 |
| Zinc | 10,35486 | 10,61841 |
| Copper | 1,237777 | 1,061133 |
| Selenium | 124,1262 | 117,0144 |
| Sodium | 3206,01 | 3194,721 |
| Potassium | 2780,615 | 2209,848 |
| ButyricAcid | 0,410859 | 0,549608 |
| CaproicAcid | 0,260328 | 0,349901 |
| CapryllicAcid | 0,213645 | 0,264777 |
| CapricAcid | 0,416222 | 0,552565 |
| LauricAcid | 0,661457 | 0,885686 |
| MyristicAcid | 1,777679 | 2,1 |
| PalmiticAcid | 14,41751 | 13,9171 |
| MargaricAcid | 0,108694 | 0,104514 |
| StearicAcid | 5,332633 | 6,303761 |
| ArachidicAcid | 0,203975 | 0,186308 |
| BehenicAcid | 0,130914 | 0,08267 |
| MyristoleicAcid | 0,078924 | 0,048696 |
| PalmitoleicAcid | 1,210174 | 1,123529 |
| OleicAcid | 28,20171 | 25,96667 |
| GadoleicAcid | 0,281064 | 0,240123 |
| ErucicAcid | 0,008925 | 0,004587 |
| LinoleicAcid | 15,13148 | 15,75218 |
| LinolenicAcid | 1,399184 | 1,54321 |
| ParinaricAcid | 0 | 0 |
| ArachidonicAcid | 0,143877 | 0,140723 |
| EPA | 0,01453 | 0,015432 |
| DPA | 0,020754 | 0,018627 |
| DHA | 0,039679 | 0,041295 |
| Tryptophan | 1,122902 | 0,929817 |
| Threonine | 3,567362 | 3,029487 |
| Isoleucine | 4,131825 | 3,509138 |
| Leucine | 7,174333 | 5,902087 |
| Lysine | 6,102763 | 5,181347 |
| Methionine | 2,035182 | 1,789845 |
| Cystine | 1,296534 | 1,041414 |
| Phenylalanine | 4,106853 | 3,295692 |

**Abbreviations**. ePOR = endoscopic postoperative recurrence

| **Micro- or macronutrients** | **Median value in no ePOR group** | **Median value in ePOR group** |
| --- | --- | --- |
| Tyrosine | 3,159529908 | 2,677461 |
| Valine | 4,616653178 | 3,859174 |
| Arginine | 4,974228056 | 4,06788 |
| Histidine | 2,590165759 | 2,036164 |
| Alanine | 4,211549502 | 3,607945 |
| AsparticAcid | 8,030034083 | 6,935652 |
| GlutamicAcid | 18,19883718 | 14,57342 |
| Glycine | 3,864265674 | 3,171848 |
| Proline | 5,734291833 | 5,210092 |
| Serine | 4,156094551 | 3,446891 |
| Aspartame | 0 | 0 |
| Saccharin | 0 | 0 |
| Caffeine | 95,85299 | 86,57996 |
| PhyticAcid | 575,6644 | 548,9747 |
| OxalicAcid | 158,0364 | 147,8076 |
| X3Methylhistidine | 16,73348 | 14,93196 |
| SucrosePolyester | 0 | 0 |
| Ash | 18,33275 | 16,23205 |
| Water | 1621,661 | 1477,02 |
| PercentCalfromFat | 40,92043 | 36,47926 |
| PercentCalfromCarb | 55,74413 | 50,87597 |
| PercentCalfromProtein | 20,10869 | 17,4695 |
| PercentCalfromAlcohol | 0,006719 | 0,01217 |
| PercentCalfromSFA | 12,48718 | 12,38265 |
| PercentCalfromMUFA | 13,98434 | 12,57593 |
| PercentCalfromPUFA | 9,155361 | 7,695565 |
| PUFtoSatFatRatio | 0,834538 | 0,74321 |
| CholtoSatFarIndex | 41,74422 | 41,9177 |
| RetinolEquivalents | 806,3441 | 809,3618 |
| transoctadecenoicacid | 1,336811 | 1,185484 |
| transoctadecadienoicacid | 0,257703 | 0,32156 |
| transhexadecenoicacid | 0,037982 | 0,032192 |
| TotalTRANS | 1,771568 | 1,660606 |
| BetacaroteneProvataminACarotenoid | 1656,994 | 1160,362 |
| AlphacaroteneProvataminACarotenoid2 | 86,52748 | 37,07033 |
| BetaCryptoxanthin | 35,58205 | 14,20052 |
| LuteinandZeaxanthin | 903,4137 | 741,3271 |
| Lycopene | 1998,925 | 1744,007 |
| FolateEquivalents | 526,6627 | 532,9153 |
| FoodFolate | 221,4461 | 190,9495 |

**Abbreviations**. ePOR = endoscopic postoperative recurrence; NA = not applicable

| **Micro- or macronutrients** | **Median value in no ePOR group** | **Median value in ePOR group** |
| --- | --- | --- |
| SyntheticFolicAcid | 139,0237 | 182,9765 |
| TotalvitA.Activity | 676,3274 | 721,3359 |
| EnergykiloJ | 9386,93 | 9146,68 |
| NiacinEquiv | 46,49568 | 38,60784 |
| TotalSugars | 95,15758 | 102,7901 |
| Omega3FA | 1,655485 | 1,607187 |
| Manganese | 3,629333 | 3,158025 |
| VitaminE2 | 15,46365 | 15,25889 |
| DAlpaTocopherol | 9,585066 | 9,667408 |
| SynthAlphaTocopherol | 0 | 0 |
| Daidzein | 0,127491 | 0,080864 |
| Genistein | 0,132929 | 0,068 |
| Glycitein | 0 | 0 |
| Coumestrol | 0,044564 | 0,002823 |
| Biochanin.A | 0 | 0 |
| Formononetin | 0 | 0 |
| AddedSugars | 56,70185 | 64,02593 |
| AcesulfamePotassium | 0 | 0 |
| Sucralose | 0 | 0 |
| AvailableCarbohydrate | 225,1329 | 214,7713 |
| GlycaemicIndex | 71,686 | 68,2044 |
| GIBread | 102,4881 | 97,51648 |
| GlycaemicLoad | 131,0308 | 135,6423 |
| GlycaemicLoadBread | 187,3182 | 193,8851 |
| Choline | 346,2778 | 388,0912 |
| Betaine | 125,1245 | 134,8729 |
| Erythritol | 0 | 0 |
| Inositol | 0,181232 | 0,115432 |
| Isomalt | 0,145641 | 0,137289 |
| Lactitol | 0 | 0 |
| Maltitol | 0,05228 | 0,026682 |
| Mannitol | 0,012732 | 0,007546 |
| Pinitol | 15,40168 | 12,46279 |
| Sorbitol | 0,095266 | 0,11358 |
| Xylitol | 0,079691 | 0,09321 |
| Nitrogen | 0,015046 | 0,01743 |
| TotalConjLinoleicAcid | 0,047311 | 0,244231 |
| CLAcis9trans11 | 139,0237 | 182,9765 |
| CLAtrans10cis11 | 676,3274 | 721,3359 |
| Tagatose | 9386,93 | 9146,68 |

**Abbreviations**. ePOR = endoscopic postoperative recurrence; NA = not applicable

| **Micro- or macronutrients** | **Median value in no ePOR group** | **Median value in ePOR group** |
| --- | --- | --- |
| Ergocalciferol | 0 | 0 |
| Cholecalciferol | 4,47941 | 3,918824 |
| AddedSugars2 | 50,65537 | 52,92752 |
| TotalGrains | 6,673051 | 6,462986 |
| WholeGrains | 0,426647 | 0,635679 |
| RefinedGrains | 5,618539 | 5,885321 |
| Alphalinoleicacid | 1,344322 | 1,522305 |
| SolidFats | 28,39125 | 30,87075 |

**Abbreviations**. ePOR = endoscopic postoperative recurrence

**Supplementary Table 2.** Nutrient analysis of all assessed macro- and micronutrients (*mean log values*)

| **Micro- or macronutrients** | **Mean log value in no ePOR group** | **Mean log value in ePOR group** | **p-value** |
| --- | --- | --- | --- |
| kcal | 7,652723 | 7,615896 | 0,634301 |
| TotalFat | 4,406692 | 4,37758 | 0,758584 |
| TotalCarbohydrate | 5,476087 | 5,489559 | 0,874145 |
| TotalProtein | 4,477977 | 4,390253 | 0,301125 |
| AnimalProtein | 3,969874 | 3,976989 | 0,950949 |
| VegProtein | 3,37268 | 3,251166 | 0,207597 |
| Alcohol | 0,441408 | 0,548959 | 0,608174 |
| Cholesterol | 5,571047 | 5,614407 | 0,73924 |
| SFA | 3,270765 | 3,296015 | 0,805399 |
| MUFA | 3,417777 | 3,329794 | 0,357421 |
| PUFA | 2,929806 | 2,899062 | 0,761333 |
| Fructose | 2,850848 | 2,922947 | 0,643299 |
| Galactose | 0,245732 | 0,313622 | 0,447123 |
| Glucose | 2,899404 | 2,937616 | 0,780077 |
| Lactose | 1,953004 | 1,843708 | 0,549295 |
| Maltose | 1,210401 | 1,118573 | 0,430828 |
| Sucrose | 3,688527 | 3,757088 | 0,604985 |
| Starch | 4,612779 | 4,645475 | 0,765363 |
| TotalFiber | 2,961489 | 2,86411 | 0,304172 |
| SolFibre | 1,966101 | 1,928353 | 0,658937 |
| InsolFibre | 2,556439 | 2,432174 | 0,216184 |
| Pectins | 1,234182 | 1,062085 | 0,066893 |
| VitAActivity | 8,587673 | 8,402971 | 0,289209 |
| BetaCaroteneEquivalents | 7,479532 | 6,970732 | 0,062944 |
| Retinol | 6,016024 | 6,165473 | 0,359337 |
| Calciferol | 1,755622 | 1,571187 | 0,14299 |
| AlphaTocopherol | 2,554219 | 2,626388 | 0,518082 |
| VitaminE | 2,388296 | 2,395028 | 0,950033 |
| BetaTocopherol | 0,404045 | 0,411538 | 0,865975 |
| GammaTocopherol | 2,587334 | 2,636388 | 0,685802 |
| DeltaTocopherol | 1,334114 | 1,327161 | 0,948933 |
| VitaminK | 4,481003 | 4,437545 | 0,785615 |
| VitaminC | 4,026717 | 4,040917 | 0,937915 |
| VitaminB1 | 1,045097 | 0,998225 | 0,379451 |
| VitamonB2 | 1,179138 | 1,133159 | 0,387017 |
| VitaminB3 | 3,325719 | 3,245243 | 0,321515 |
| PantothenicAcid | 1,894703 | 1,852643 | 0,529487 |
| VitaminB6 | 1,129361 | 1,074264 | 0,345231 |
| Folate | 5,987037 | 5,965288 | 0,802144 |
| VitaminB12 | 1,633026 | 1,620527 | 0,911986 |

**Abbreviations**. ePOR = endoscopic postoperative recurrence

| **Micro- or macronutrients** | **Mean log value in no ePOR group** | **Mean log value in ePOR group** | **p-value** |
| --- | --- | --- | --- |
| Calcium | 6,7413 | 6,764948 | 0,815748 |
| Phosphorus | 7,157461 | 7,124882 | 0,675901 |
| Magnesium | 5,719006 | 5,57486 | 0,105019 |
| Iron | 2,776524 | 2,730831 | 0,592778 |
| Zinc | 2,409655 | 2,413981 | 0,95652 |
| Copper | 0,827013 | 0,80802 | 0,763645 |
| Selenium | 4,831622 | 4,756954 | 0,371938 |
| Sodium | 8,042379 | 8,079288 | 0,671523 |
| Potassium | 7,885291 | 7,75009 | 0,119795 |
| ButyricAcid | 0,371558 | 0,454427 | 0,101653 |
| CaproicAcid | 0,250416 | 0,306952 | 0,113372 |
| CapryllicAcid | 0,234411 | 0,302113 | 0,142414 |
| CapricAcid | 0,395972 | 0,486904 | 0,091421 |
| LauricAcid | 0,596626 | 0,752687 | 0,146282 |
| MyristicAcid | 1,094316 | 1,2319 | 0,124097 |
| PalmiticAcid | 2,700111 | 2,67391 | 0,774347 |
| MargaricAcid | 0,12033 | 0,130138 | 0,573868 |
| StearicAcid | 1,952662 | 1,934203 | 0,844991 |
| ArachidicAcid | 0,190778 | 0,17399 | 0,419909 |
| BehenicAcid | 0,175016 | 0,142952 | 0,32624 |
| MyristoleicAcid | 0,096241 | 0,111911 | 0,591089 |
| PalmitoleicAcid | 0,773587 | 0,739918 | 0,547201 |
| OleicAcid | 3,351481 | 3,269046 | 0,38832 |
| GadoleicAcid | 0,292228 | 0,24636 | 0,153139 |
| ErucicAcid | 0,030487 | 0,021956 | 0,477671 |
| LinoleicAcid | 2,796648 | 2,775453 | 0,833199 |
| LinolenicAcid | 0,945626 | 0,995457 | 0,496109 |
| ParinaricAcid | 0,01327 | 0,00284 | 0,029178 |
| ArachidonicAcid | 0,177723 | 0,152571 | 0,300137 |
| EPA | 0,05644 | 0,036508 | 0,248549 |
| DPA | 0,03593 | 0,027518 | 0,306792 |
| DHA | 0,124275 | 0,0757 | 0,144422 |
| Tryptophan | 0,737496 | 0,694325 | 0,312748 |
| Threonine | 1,484406 | 1,419283 | 0,324217 |
| Isoleucine | 1,593571 | 1,529878 | 0,348134 |
| Leucine | 2,049595 | 1,97906 | 0,332938 |
| Lysine | 1,900396 | 1,832052 | 0,390732 |
| Methionine | 1,094499 | 1,052043 | 0,450732 |
| Cystine | 0,792985 | 0,739004 | 0,205513 |
| Phenylalanine | 1,592506 | 1,520094 | 0,258373 |

**Abbreviations**. ePOR = endoscopic postoperative recurrence

| **Micro- or macronutrients** | **Mean log value in no ePOR group** | **Mean log value in ePOR group** | **p-value** |
| --- | --- | --- | --- |
| Tyrosine | 1,398856 | 1,345338 | 0,396852 |
| Valine | 1,691976 | 1,62534 | 0,328578 |
| Arginine | 1,755323 | 1,653082 | 0,183465 |
| Histidine | 1,225112 | 1,171618 | 0,378068 |
| Alanine | 1,626204 | 1,557126 | 0,330245 |
| AsparticAcid | 2,161523 | 2,075446 | 0,282219 |
| GlutamicAcid | 2,8996 | 2,81117 | 0,266744 |
| Glycine | 1,531147 | 1,443396 | 0,22051 |
| Proline | 1,877517 | 1,837722 | 0,571734 |
| Serine | 1,618409 | 1,542819 | 0,231736 |
| Aspartame | 0,181515 | 0,538923 | 0,20774 |
| Saccharin | 0 | 0 | NA |
| Caffeine | 4,046511 | 3,987795 | 0,870343 |
| PhyticAcid | 6,398465 | 6,244622 | 0,213516 |
| OxalicAcid | 5,107664 | 4,993119 | 0,368667 |
| X3Methylhistidine | 2,690281 | 2,600983 | 0,654749 |
| SucrosePolyester | 0 | 0 | NA |
| Ash | 2,922774 | 2,884931 | 0,602366 |
| Water | 7,29671 | 7,308863 | 0,892776 |
| PercentCalfromFat | 3,697127 | 3,642255 | 0,341658 |
| PercentCalfromCarb | 3,97079 | 3,962614 | 0,891282 |
| PercentCalfromProtein | 2,991306 | 2,897512 | 0,161914 |
| PercentCalfromAlcohol | 0,282437 | 0,346323 | 0,653928 |
| PercentCalfromSFA | 2,58082 | 2,583558 | 0,964871 |
| PercentCalfromMUFA | 2,727407 | 2,616179 | 0,060061 |
| PercentCalfromPUFA | 2,260575 | 2,201301 | 0,421454 |
| PUFtoSatFatRatio | 0,62658 | 0,601119 | 0,590548 |
| CholtoSatFarIndex | 3,722271 | 3,742459 | 0,839916 |
| RetinolEquivalents | 6,806672 | 6,734349 | 0,625843 |
| transoctadecenoicacid | 0,900223 | 0,906543 | 0,94855 |
| transoctadecadienoicacid | 0,278937 | 0,284494 | 0,854675 |
| transhexadecenoicacid | 0,045203 | 0,050115 | 0,585884 |
| TotalTRANS | 1,047822 | 1,056728 | 0,927703 |
| BetacaroteneProvataminACarotenoid | 7,37289 | 6,889564 | 0,07583 |
| AlphacaroteneProvataminACarotenoid2 | 4,701157 | 3,752835 | 0,024759 |
| BetaCryptoxanthin | 3,449023 | 3,099819 | 0,204125 |
| LuteinandZeaxanthin | 6,988766 | 6,735754 | 0,284348 |
| Lycopene | 7,008686 | 6,785424 | 0,681132 |
| FolateEquivalents | 6,242564 | 6,228306 | 0,877992 |
| FoodFolate | 5,357506 | 5,27362 | 0,416771 |

**Abbreviations**. ePOR = endoscopic postoperative recurrence; NA = not applicable

| **Micro- or macronutrients** | **Mean log value in no ePOR group** | **Mean log value in ePOR group** | **p-value** |
| --- | --- | --- | --- |
| SyntheticFolicAcid | 4,939274 | 4,904582 | 0,868357 |
| TotalvitA.Activity | 6,543179 | 6,534354 | 0,950813 |
| EnergykiloJ | 9,083595 | 9,046765 | 0,634406 |
| NiacinEquiv | 3,822439 | 3,743717 | 0,330651 |
| TotalSugars | 4,513435 | 4,572154 | 0,584971 |
| Omega3FA | 1,034813 | 1,035008 | 0,99804 |
| Manganese | 1,527574 | 1,475208 | 0,498122 |
| VitaminE2 | 2,758203 | 2,794744 | 0,753122 |
| DAlpaTocopherol | 2,360394 | 2,278452 | 0,455166 |
| SynthAlphaTocopherol | 0,129668 | 0,490567 | 0,054066 |
| Daidzein | 0,460734 | 0,154799 | 0,006104 |
| Genistein | 0,510538 | 0,147613 | 0,00346 |
| Glycitein | 0,161477 | 0,024813 | 0,0118 |
| Coumestrol | 0,071005 | 0,053474 | 0,316281 |
| Biochanin.A | 0,044208 | 0,038129 | 0,859823 |
| Formononetin | 0,001729 | 0,001298 | 0,566598 |
| AddedSugars | 3,970511 | 4,035015 | 0,713837 |
| AcesulfamePotassium | 0,147252 | 0,288409 | 0,413238 |
| Sucralose | 0,261519 | 0,320836 | 0,779641 |
| AvailableCarbohydrate | 5,38421 | 5,405778 | 0,811189 |
| GlycaemicIndex | 4,252971 | 4,216159 | 0,441569 |
| GIBread | 4,605755 | 4,568924 | 0,444598 |
| GlycaemicLoad | 4,878811 | 4,919484 | 0,668046 |
| GlycaemicLoadBread | 5,233483 | 5,274392 | 0,667151 |
| Choline | 5,838058 | 5,805819 | 0,714304 |
| Betaine | 4,828694 | 4,85969 | 0,80217 |
| Erythritol | 0,006855 | 0,054957 | 0,223734 |
| Inositol | 0,209379 | 0,123388 | 0,002854 |
| Isomalt | 0 | 0 | NA |
| Lactitol | 0 | 0 | NA |
| Maltitol | 0 | 0 | NA |
| Mannitol | 0,243359 | 0,181393 | 0,152096 |
| Pinitol | 0,024897 | 0,00609 | 0,024611 |
| Sorbitol | 0,195054 | 0,168605 | 0,612137 |
| Xylitol | 0,017257 | 0,010612 | 0,028806 |
| Nitrogen | 2,724109 | 2,638507 | 0,278306 |
| TotalConjLinoleicAcid | 0,112236 | 0,125242 | 0,44326 |
| CLAcis9trans11 | 0,094479 | 0,106048 | 0,416077 |
| CLAtrans10cis11 | 0,019146 | 0,02218 | 0,451979 |
| Tagatose | 0,284732 | 0,336806 | 0,649232 |

**Abbreviations**. ePOR = endoscopic postoperative recurrence; NA = not applicable

| **Micro- or macronutrients** | **Mean log value in no ePOR group** | **Mean log value in ePOR group** | **p-value** |
| --- | --- | --- | --- |
| Ergocalciferol | 0,173104 | 0,189528 | 0,845319 |
| Cholecalciferol | 1,67772 | 1,506015 | 0,173914 |
| AddedSugars2 | 3,868539 | 3,956424 | 0,614296 |
| TotalGrains | 2,061239 | 2,060266 | 0,992344 |
| WholeGrains | 0,57767 | 0,539868 | 0,748139 |
| RefinedGrains | 1,893568 | 1,845486 | 0,702557 |
| Alphalinoleicacid | 0,922933 | 0,975813 | 0,471294 |
| SolidFats | 3,471823 | 3,501965 | 0,810033 |

**Abbreviations**. ePOR = endoscopic postoperative recurrence

**Supplementary Table 3**. Variable importance of the random forest models including the clinical risk factors (*upper*), identified nutrients (*middle*) and clinical risk factors and nutrients combined (*below*)

| **Variable** | **Variable importance** |
| --- | --- |
| Inositol | 0.1732 |
| Provitamin-A Carotenoid | 0.1722 |
| Xylitol | 0.1588 |
| Genistein | 0.1528 |
| Daidzein | 0.1523 |
| Parinaric acid | 0.1067 |
| Pinitol | 0.0521 |
| Glycitein | 0.0321 |

| **Variable** | **Variable importance** |
| --- | --- |
| Age at surgery | 0.3532 |
| Disease duration | 0.2911 |
| Active smoking | 0.0728 |
| Postoperative prophylactic medication | 0.0694 |
| Perianal disease (Montreal p+) | 0.0682 |
| Sex | 0.0664 |
| Penetrating disease (Montreal B3) | 0.0521 |
| Prior small bowel resection | 0.0267 |

| **Variable** | **Variable importance** |
| --- | --- |
| Age at surgery | 0.1287 |
| Provitamin-A Carotenoid | 0.1213 |
| Xylitol | 0.1082 |
| Inositol | 0.1071 |
| Disease duration at surgery | 0.1018 |
| Genistein | 0.0934 |
| Daidzein | 0.0897 |
| Parinaric acid | 0.0728 |
| Active smoking | 0.0388 |
| Pinitol | 0.0318 |
| Postoperative prophylactic medication | 0.0249 |
| Sex | 0.0232 |
| Perianal disease (Montreal p+) | 0.0209 |
| Glycitein | 0.0191 |
| Penetrating disease (Montreal B3) | 0.0114 |
| Prior small bowel resection | 0.0068 |
